# An overview of host‐derived molecules that interact with gut microbiota

**DOI:** 10.1002/imt2.88

**Published:** 2023-02-13

**Authors:** Chenguang Zhang, Huifeng Liu, Lei Sun, Yue Wang, Xiaodong Chen, Juan Du, Åsa Sjöling, Junhu Yao, Shengru Wu

**Affiliations:** ^1^ College of Animal Science and Technology Northwest A&F University Yangling China; ^2^ Centre for Translational Microbiome Research, Department of Microbiology, Tumor and Cell Biology Karolinska Institutet Stockholm Sweden

**Keywords:** exosomal ncRNA, gut mucosal molecules, gut‐derived immune molecules, hormones, host, microbiota shaping, molecules from other organs than gut

## Abstract

The gut microbiota comprises bacteria, archaea, fungi, protists, and viruses that live together and interact with each other and with host cells. A stable gut microbiota is vital for regulating host metabolism and maintaining body health, while a disturbed microbiota may induce different kinds of disease. In addition, diet is also considered to be the main factor that influences the gut microbiota. The host could shape the gut microbiota through other factors. Here, we reviewed the mechanisms that mediate host regulation on gut microbiota, involved in gut‐derived molecules, including gut‐derived immune system molecules (secretory immunoglobulin A, antimicrobial peptides, cytokines, cluster of differentiation 4^+^ effector T cell, and innate lymphoid cells), sources related to gut‐derived mucosal molecules (carbon sources, nitrogen sources, oxygen sources, and electron respiratory acceptors), gut‐derived exosomal noncoding RNA (ncRNAs) (microRNAs, circular RNA, and long ncRNA), and molecules derived from organs other than the gut (estrogen, androgen, neurohormones, bile acid, and lactic acid). This study provides a systemic overview for understanding the interplay between gut microbiota and host, a comprehensive source for potential ways to manipulate gut microbiota, and a solid foundation for future personalized treatment that utilizes gut microbiota.

## Highlights

- The host‐derived molecules that could interact with the gut microbiota and the mechanism of how these molecules affected the gut microbiota were summarized.
- The host‐derived molecules that shape the gut microbiota include gut‐derived immune molecules, sources related to gut‐derived mucosal molecules, gut‐derived exosomal noncoding RNAs, and molecules derived from organs other than the gut were separately reviewed.
- Understanding how host factors regulate the gut microbiota and influence disease incidence can help to develop novel preventive and therapeutic interventions, improve the cure rate of fecal microbiota transplantation, and even aid in the prediction of disease susceptibility in individuals.

## Graphical abstract

The mechanisms that mediate host regulation on gut microbiota, involved in the gut‐ and nongut‐derived metabolites, including gut‐derived immune system factors, sources related to gut‐derived mucosal metabolites, gut‐derived exosomes' noncoding RNA regulation, and nongut‐derived metabolite‐related sources are discussed to provide a systematic overview of how host metabolites govern the gut microbiota.

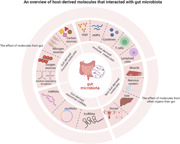

## INTRODUCTION

The multiple roles of the microbiota, comprising bacteria, archaea, fungi, protists, and viruses, in food digestion, modifying drug actions, neurological signaling, metabolic processes, training host immunity, and regulating gut endocrine function have recently gained increased attention [1, 2]. Several signaling connections, such as the gut–kidney, gut–liver, and gut–brain axes, have been identified and proved to be involved in the processes, where the gut microbiota affect host phenotypes through either the directed interaction between bacteria and differential intestinal cells or the indirect interaction through the microbial composition or their molecules [3, 4]. With the development of high‐throughput sequencing technologies and the establishment of sterile pig models, germ‐free mice, and gene knockout mice, the importance of how the gut microbiota influence health and disease in the host have gradually increased in recognition. Large arrays of molecules have been proven to drive the crosstalk between the host and its microbiome, especially the single‐direction regulation of microbiota in shaping host phenotype changes [5]. In contrast, studies focused on mechanisms where host‐derived molecules directly shape the gut microbiota are worthy of more attention.

The microbiota of the gut is suggested to be shaped by host genetics, diet, geography, social contact patterns, and so on [6]. Of these factors, diet is a primary driver that is widely proposed to directly influence the gut microbial ecosystem [7]. Recently, an increasing number of studies have found that individuals can have different gut microbiota‐regulated physiological responses under the same dietary or environmental conditions, hinting that the difference could be attributed to the host effects [8]. Further, the intergenerational inheritance of gut microbiota, as well as the link between the host genetics and gut microbiota composition [9], has indicated the existence and importance of the potential ways host factors shape the gut microbiota.

Despite increasing unambiguous evidence supporting that the host shapes the gut microbiota, the underlying mechanisms are rarely discussed and systematically reviewed. Notably, microbiome genome‐wide association studies (mGWAS) have identified a series of genes associated with the colonization of the microbiota [10]. However, the underlying mechanism focused on the identified host‐derived molecules from the intestinal epithelium on the gut microbiota, while the mechanism behind the direct effects was especially less reviewed. Hence, the present review mainly focused on the mechanisms of host‐derived molecules that interacted with and shaped the gut microbiome. Further, these related mechanisms were divided into four parts: (1) gut‐derived immune system molecules, (2) sources related to gut‐derived mucosal molecules, (3) gut‐derived exosomal noncoding RNA (ncRNA) regulation, and (4) molecules derived from organs other than the gut. In short, the aim of this review is to systematically examine the mechanisms of host‐derived molecules regulating the gut microbiota and to deepen our understanding of the host–microbiota crosstalk.

## GUT‐DERIVED IMMUNE SYSTEM MOLECULES

The immune systems, including both innate and adaptive immune cells, could also be involved in the regulation of the gut microbiota. The adaptive immune system mainly responds to pathogen infestation and subsequently induces multiple cytokines in response to microbial‐derived signals to initiate inflammatory responses to remove pathogenic bacteria [11]. It was decided not to extend the present review to the truly complex network between all individual microorganisms constituting the microbiota but to rather focus on the entire microbiota.

### Secretory immunoglobulin A

Peyer's patches are an important part of intestinal mucosal immunity and produce secretory immunoglobulin A (sIgA). The sIgA is a dimer composed of IgA, joining chain (J‐chain), and secretory component (SC), in which the J‐chain links two IgA monomers together, and SC binds to IgA via disulfide bond [10]. Besides targeting invasive pathogens, sIgA widely targets noninvasive commensals. In the absence of IgA‐coating bacteria, the host will be harmfully affected [12]. Importantly, the microbial colonization of early life is a sequential process, which varies in parallel with the clonal architecture of gut sIgA. Until the stable gut microbiota of adult humans are established, sIgA polyclonal antibodies broadly cover commensals [13]. The coevolution between IgA and microbiota hence regulates microbiota at all stages of life.

**Figure 1:**
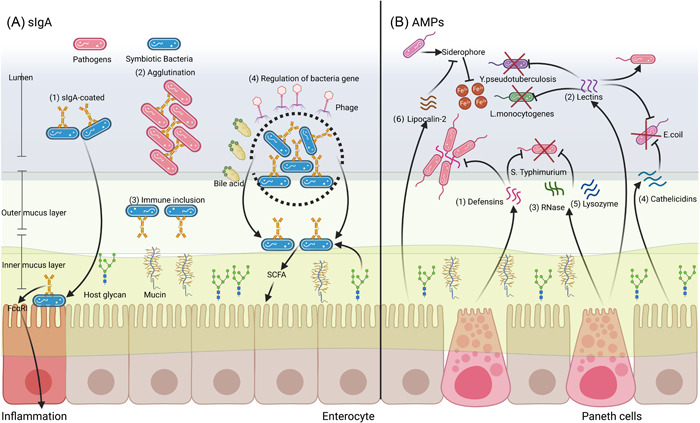
Gut‐derived immune system molecules (secretory immunoglobulin A [sIgA] and antimicrobial peptides [AMPs]) of host‐shaping gut microbiota. (A) The mechanisms of sIgA shaping gut microbiota: (1) sIgA‐coated, the sIgA‐coated commensals are confined in the lumen [14], but they could enhance inflammatory response in the mucosa layer. (2) Agglutination, the agglutination of pathogens in the lumen is promoted [15]. (3) Immune inclusion, the colonization of commensals is enhanced in the mucosa layer [16]. (4) Regulation of bacteria gene, sIgA shapes the growth and metabolic functions of bacteria by regulating bacterial gene transcription [13]. (B) The mechanisms of antimicrobial peptides shaping gut microbiota: (1) Defensins are produced by Paneth cells and can capture or kill *Salmonella enterica Typhimurium* (*S. Typhimurium*). (2) Regenerating (Reg) protein family is produced by Paneth cells and can kill *Listeria monocytogenes* [17], *Enterococcus* [18], and *Yersinia pseudotuberculosis* [19], but prolong the infections with *S. Typhimurium* [20]. (3) Ribonuclease (RNase) angiogenin 4 (Ang4) is produced by Paneth cells and can kill *S. Typhimurium* [21]. (4) Cathelicidins are produced by enterocytes and could kill *Escherichia coli* [22]. (5) Lysozymes are produced by Paneth cells and can kill *S. Typhimurium* [23]. (6) Lipocalin‐2 is produced by enterocytes and can quarantine the siderophore from *E. coli* [24].

**Figure 2:**
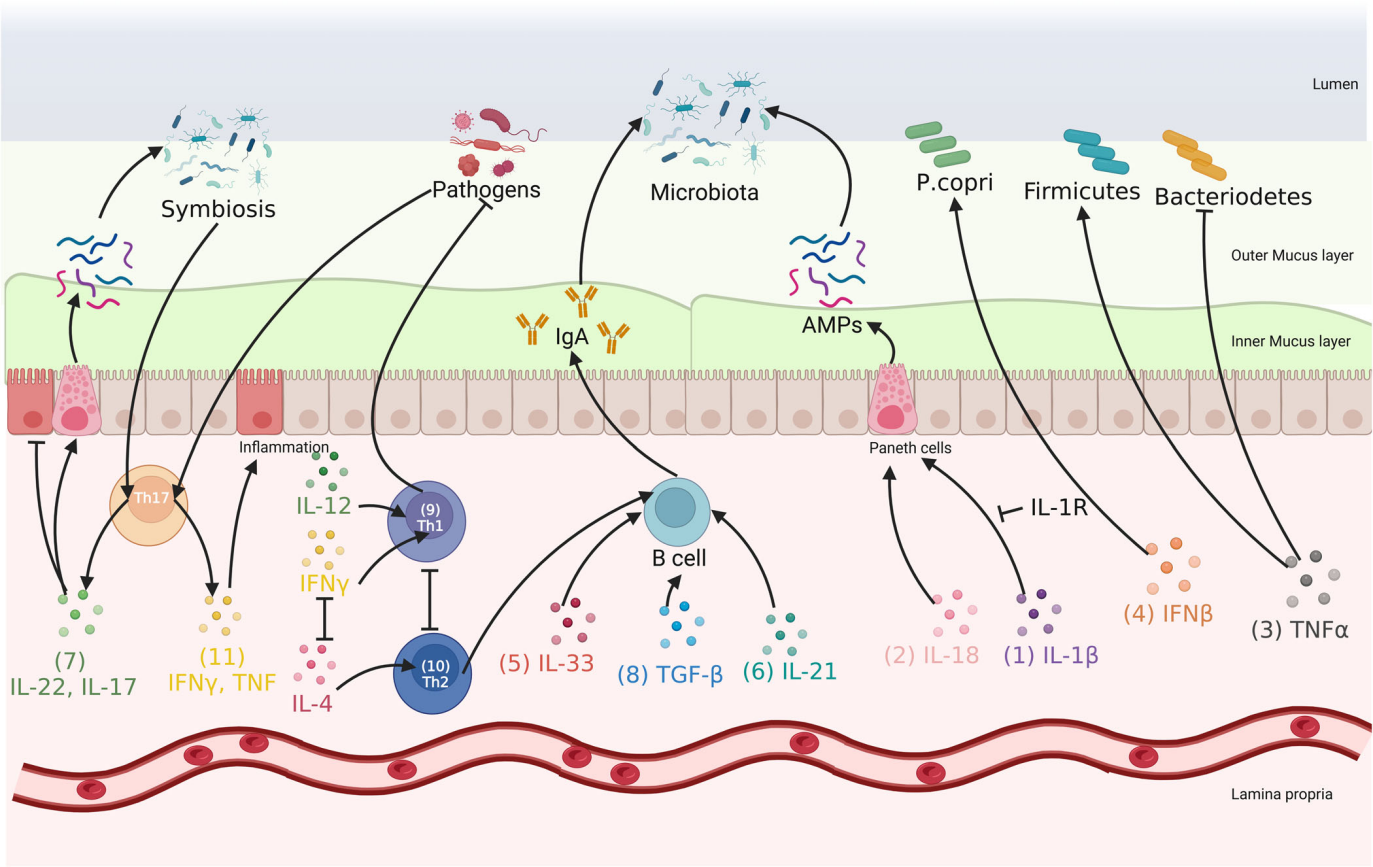
Gut‐derived immune system molecules (cytokine) of host shaping gut microbiota. The mechanisms of cytokine shaping gut microbiota: (1) interleukin‐1β (IL‐1β) could regulate gut microbiota by promoting the production of antimicrobial peptides (AMPs), and the IL‐1R could suppress the process [41]. (2) IL‐1 and IL‐18 could also regulate the gut microbiota by promoting the production of AMPs [42]. (3) Tumor necrosis factor‐α (TNF‐α) could reduce the Firmicutes to Bacteroidetes ratio [43]. (4) Interferon‐β (IFN‐β) could increase the abundance of *Prevotella copri* [44]. (5, 6, 8) IL‐33 and IL‐21 and transforming growth factor‐β (TGF‐β) could promote the production by modulating B‐cell differentiation [45, 46, 47]. (7) IL‐17 and IL‐22 could maintain mucosal integrity [48]. (9) IFN‐γ and IL‐12 initiate the differentiation of T‐helper type (Th1) cells and accelerate the removal of pathogens [49]. (10) IL‐4 initiates the differentiation of Th2 cells and is key in helping B cells to produce antibodies [49]. (7, 11) Th17 cells produce different cytokines according to the kind of gut microbiota [50].

**Figure 3:**
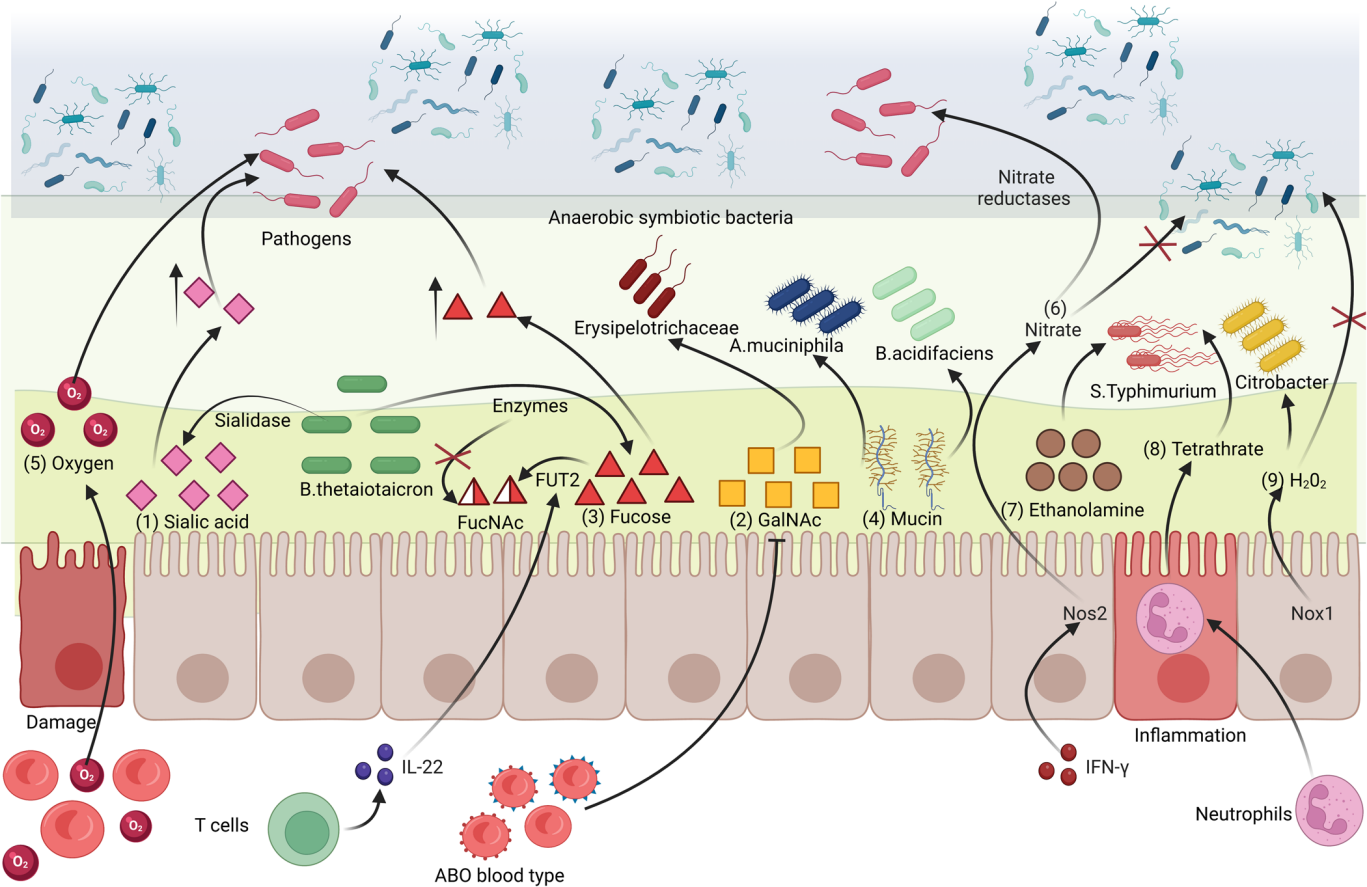
Sources related to gut‐derived mucosal molecules shaping the gut microbiota. The mechanisms of host‐derived nutrient source shaping gut microbiota: (1) Sialic acid is released into the lumen by *Bacteroides thetaiotaomicron* and could be utilized by pathogens [63]. (2) *N*‐acetyl‐galactosamine could be utilized by Erysipelotrichaceae [64]. (3) Fucose is released into the lumen by *B. thetaiotaomicron* and could be utilized by *Salmonella enterica Typhimurium* (*S. Typhimurium*) [63], which could be inhibited by glycosylation [63]. (4) Mucins could be utilized by *Akkermansia muciniphila* and *Bacteroides acidifaciens* [65]. (5) The leakage of the epithelium will lead to an increase in the luminal oxygen level, which could provide an ecological selective advantage to facultative anaerobes or potential aerobes [66]. (6) Nitrate could be produced by *Nos2* gene of enterocytes and could be utilized by pathogens [67]. (7) Ethanolamine and (8) tetrathionate could be utilized by *S. Typhimurium* [68]. (9) Hydrogen peroxide could be produced by *Nox1* gene of enterocytes and could be utilized by *Citrobacter*, but it inhibits the growth of anaerobic commensals in the mucosae layer during homeostasis [69].

**Figure 4:**
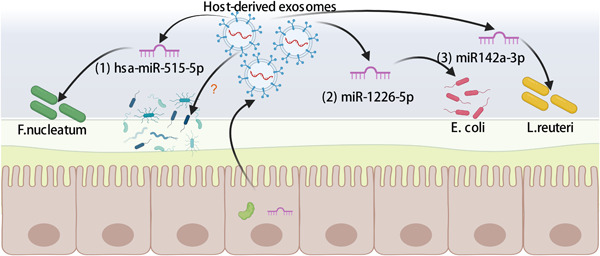
Gut‐derived exosomal shaping gut microbiota. The mechanisms of host‐derived exosomes shaping gut microbiota: (1) hsa‐miR‐515‐5p could promote the growth of *Fusobacterium nucleatum* [81], (2) miR‐1226‐5p could promote the growth of *Escherichia coil* [81], and (3) miR142a‐3p could promote the growth of *Lactobacillus reuteri* [82].

sIgA, directed to specific bacterial antigens (e.g., lipopolysaccharide [LPS] and flagella), is essential for maintaining the homeostasis of the gut microbiota. Under normal physiological conditions, sIgA regulates the gut microbiota through four main ways: (1) sIgA‐coated commensals (Figure 1A,(1)): by confining sIgA‐coated commensals in the lumen of the gut, immune exclusion minimizes proinflammatory interactions of the gut immune system with a myriad of microorganism‐associated molecular patterns, which avoid activation of the complement cascade [14]. Remarkably, sIgA can elicit inflammation by cross‐linking the Fcα receptor I on proinflammatory phagocytes following the penetration of sIgA‐coated commensals into the gut mucosa [25]. (2) Agglutination (Figure 1A,(2)): sIgA promotes the agglutination of gut commensals, which limits their motility [26]. For pathogens, high‐avidity sIgA could cross‐link daughter cells of the host to prevent the separation and spread of pathogens [15]. (3) Immune inclusion (Figure 1A,(3)): sIgA‐coated commensals are anchored to the outer mucous layer lining the epithelial surface through a mechanism involving glycans from the SC of sIgA [16]. (4) Regulation of bacterial gene expression (Figure 1A,(4)): the mucus‐associated functional factor (*MAFF*) gene could upregulate the polysaccharide utilization activity of symbiotic bacteria and increase the colonization of butyrate‐producing bacteria [27]. The change of beneficial metabolites such as butyrate could directly stimulate the proliferation and regeneration of colonic epithelial cells to deal with epithelial injury [27]. Moreover, a single microbe provides a series of sIgA‐mediated parallel mechanisms, including metabolic modulation, protection from bile acids (BAs) or phage, and motility alterations [28]. Notably, sIgA shapes the topography, composition, growth, invasiveness, and immune metabolic functions of bacteria by regulating bacterial gene transcription [13]. Finally, regulatory T‐regulatory (Treg) cell‐dominated IgA responses expand beneficial Firmicutes bacteria while constraining potentially harmful Proteobacteria, thereby modulating the composition of the gut microbiota [29].

### Antimicrobial peptides

Compared with high‐affinity sIgA, the host‐derived antimicrobial peptides (AMPs), which are expressed by multiple epithelial cells (e.g., in the rumen, intestine, lung, genital tract, etc.), exhibit broad‐spectrum antimicrobial activity [30]. Although AMPs are a diverse group of molecules in terms of sequence, structure, and sources, almost all AMPs have several properties in common. AMPs contain a specific cationic domain, which could be attributed to the presence of lysine and arginine (and sometimes histidine) residues, and lead AMPs generally display a net positive charge. Moreover, the AMPs generally contain the hydrophobic residues in the peptide sequence (typically 50% for AMPs). Finally, the balance between the cationic and hydrophobic residues determines amphipathicity, which makes AMPs have both polar and nonpolar sides [31].

Host‐derived AMPs control the microbiota and defend against pathogenic infection by disrupting bacterial membranes and sequestering essential nutrients [32]. In the gastrointestinal tract, the host‐derived AMPs mainly originate from enterocytes or Paneth cells, which are specialized epithelial cells that are positioned in the small intestinal crypts, in close proximity to the stem cells from which all the distinct lineages of gut epithelial cells originate [33]. Hence, the disturbance of the host intestinal epithelium directly affects the secretion of AMPs and the homeostasis of the gut microbiota.

**Figure 5:**
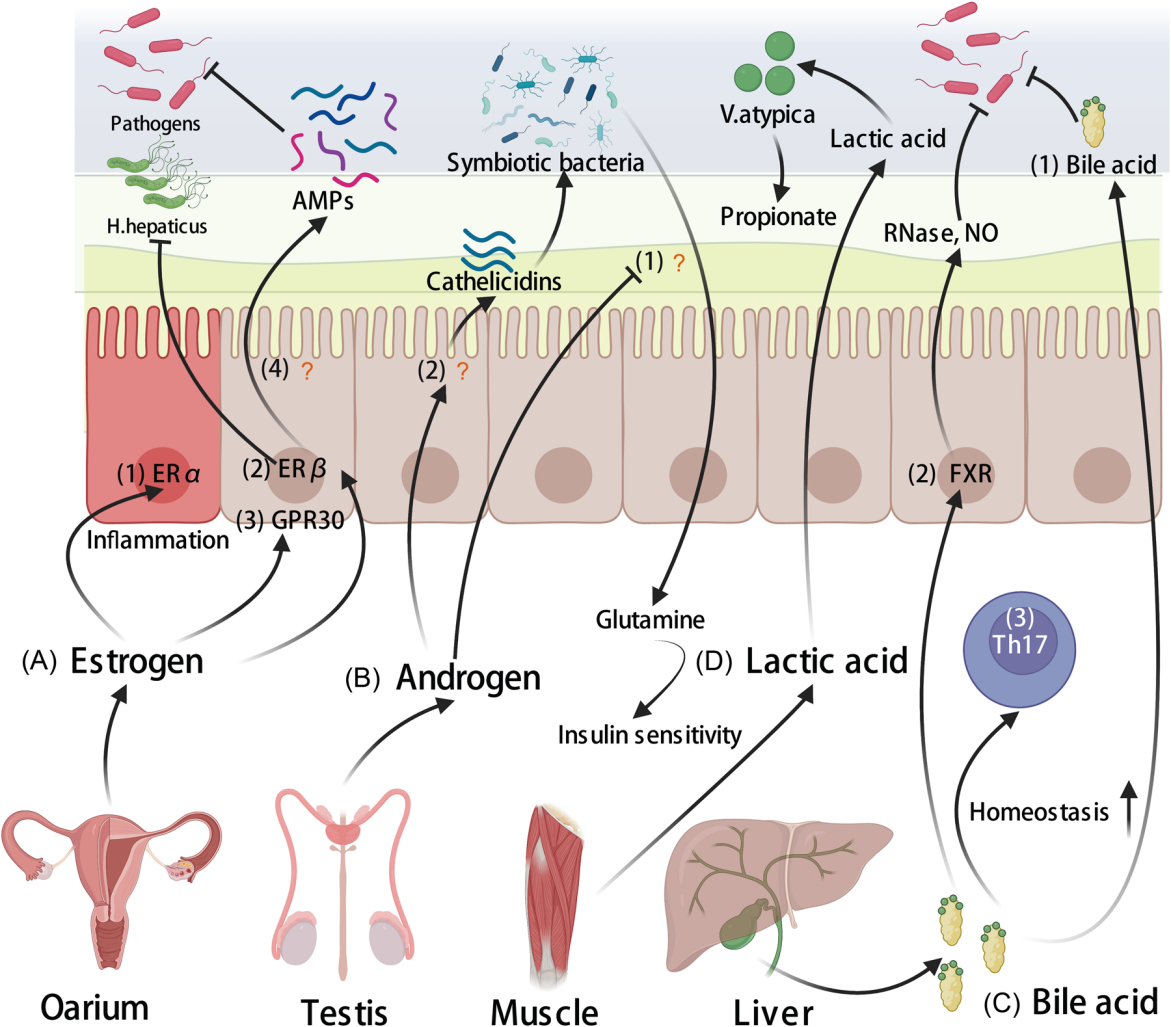
Molecules derived from other organs than the gut‐shaping gut microbiota. (A) The mechanisms of the female reproductive system shaping gut microbiota: (1) Estrogen receptor‐α (ERα) could enhance inflammatory response [92]. (2) ERβ could inhibit *Helicobacter hepaticus* [93] and regulate differentiation, tight‐junction formation, and the permeability of enterocytes [94]. (3) GPR30 could increase colonic motility and may counteract inflammation [95]. (4) The regulation of intestinal estrogen receptors on antimicrobial peptide synthesis is not clear. (B) The mechanisms of the male reproductive system shaping gut microbiota: (1) The regulation of androgen on gut microbial metabolism is not clear. (2) The regulation of androgen on antimicrobial peptide synthesis is not clear. (C) The mechanisms of bile acid shaping gut microbiota: (1) The direct effect of bile acids on gut microbiota [96]. (2) The effect of bile acids on gut microbiota through farnesoid X receptor (FXR) [97]. (3) The effect of bile acids on the homeostasis of T‐helper type 17 (Th17) cells [98]. (D) The mechanism of lactic acid shaping gut microbiota: Lactic acid is produced by muscle and could promote the growth of *Veillonella atypica* [99].

The host‐derived AMPs directly regulate the gut microbiota by interacting with bacterial membrane components, which could capture or kill pathogens. Such AMPs include defensins, lectins, ribonucleases (RNases), cathelicidins, and lysozymes [32]. The way the host regulates the gut microbiota using sIgA is mainly aimed at increasing the niche competitiveness of symbiotic bacteria, while AMPs increase the resistance toward pathogens. Several recent studies have found that defects in AMP responses represent an important factor leading to increased host susceptibility to infection with gut pathogens because the gut microbiota are more prone to disturbance [34]. Meanwhile, the contribution of AMPs is distinct for different pathogens as follows: (1) Defensins (Figure 1B,(1)): the defensins are produced by Paneth cells; mice that lack mature β‐defensins exhibit increased susceptibility to oral challenge with *Salmonella enterica Typhimurium* (*S. Typhimurium*) [35]. Moreover, the defensins can form nanonets to capture *S. Typhimuriu*m [36]. (2) Lectins (Figure 1B,(2)): antibacterial lectins can form hexameric pores in Gram‐positive bacterial membranes and prevent bacteria from reaching the intestinal mucus layer [37]. Moreover, the regenerating (Reg) protein family is a group of soluble lectins, and defects in Reg protein responses increase host susceptibility to intestinal infections with *Listeria monocytogenes* (*L. monocytogenes*) [17], *Enterococcus* [18], and *Yersinia pseudotuberculosis* (*Y. pseudotuberculosis*) [19], but they could prolong infections with *S. Typhimurium* [20]. (3) RNase (Figure 1B,(3)): RNases exhibit activity against both Gram‐positive and Gram‐negative pathogens in vitro. In vivo, mice with T‐cell receptor‐δ −/− (TCRδ^−/−^) express significantly reduced levels of the RNase angiogenin 4 (Ang4), which leads to an inhibition of Ang4 production following an oral challenge by *S. Typhimurium* [21]. (4) Cathelicidins (Figure 1B,(4)): Cathelicidin‐related AMP (CRAMP) expression deficiency in newborn mice was observed to cause an overabundance of *Escherichia coli* (*E. coli*) and increased susceptibility to the pancreatic autoimmune response and the development of diabetes in adults [22]. (5) Lysozyme (Figure 1B,(5)): Lysozymes are produced by Paneth cells, which could reroute lysozymes to maintain their antimicrobial capability during *S. Typhimurium* infection [23].

The indirect mechanisms for AMPs regulating the gut microbiota include the sequestration of essential nutrients. For example, siderophores, a class of iron‐binding compounds secreted by *E. coli*, could compete with the host's iron‐binding proteins for iron ions to provide essential nutrients for pathogens' growth [38]. (6) Lipocalin‐2 (Figure 1B,(6)): For counteracting bacterial siderophores, the host cell could produce lipocalin‐2 to quarantine the siderophores [24].

Except for pathogens' susceptibility, host‐derived AMPs may improve the production performance of domestic animals. A recent study identified a core rumen epithelial gene (*DEFB1*), which is a new paralog belonging to the β‐defensin family. Interestingly, the antibacterial activity of *DEFB1* is similar to monensin (an additive for improving the conversion efficiency of feedstuff) [39].

### Cytokines

Immune cytokines can be arranged into proinflammatory, anti‐inflammatory/proresolutive, and chemoattractive cytokines. The interleukin‐1β (IL‐1β), IL‐18, IL‐6, tumor necrosis factor (TNF), interferon (IFN), IL‐33, IL‐17, IL‐21, and IL‐13 were proinflammatory cytokines, and the IL‐10, IL‐22, IL‐4, and transforming growth factor‐β (TGF‐β) [11] were anti‐inflammatory cytokines. The relationship between these proinflammatory and anti‐inflammatory cytokines and the gut microbiota has been systematically discussed and reviewed [11]. Most cytokines are glycoproteins with a molecular mass of less than 25 kD and in the form of single chains [40]. The present review mainly focused on the roles of cytokines in affecting the gut microbiota. For instance, IL‐1β can activate IL‐1R type 2 and then suppress the production of AMPs in the colon and trigger the growth of proinflammatory gut microbiota (Figure 2,(1)) [41]. As a member of the IL‐1 family of cytokines, high levels of IL‐18 are associated with the production of AMPs, which indicates its role in regulating the gut microbiota (Figure 2,(2)) [42]. Further, most anti‐TNF therapies have been shown to ameliorate inflammation, in part, by modulating gut microbiota composition. For example, the inhibition of TNF‐α production was noted to reduce the Firmicutes to Bacteroidetes ratio (Figure 2,(3)) [43]. It was also found that IFN‐β can modulate the composition of the microbiota in patients diagnosed with multiple sclerosis and increase the abundance of *Prevotella copri* (*P. copri*) (Figure 2,(4)) [44], which suggests that IFN‐β could also be involved in regulating the gut microbiota. Moreover, mice deficient in IFN receptors (IFNAR1^−/−^) exclusively on intestinal epithelial cells (IECs) exhibited a significant alteration in gut microbiota composition and a greater susceptibility to tumor development in an experimental model of colitis [51]. Further, IL‐33 has been recently found to be an important regulator of the gut microbiota since IL‐33‐deficient mice present decreased colonic IgA, which is known to regulate the gut microbiota (Figures 2,(5)) [45]. IL‐21 also extends an important influence on IgA production by modulating B‐cell differentiation (Figure 2,(6)) [46]. This function indirectly affects gut microbiota modulation because IgA is responsible for controlling the levels of commensal and pathogenic bacteria in the intestine and participates in intestinal epithelium homeostasis [52]. Meanwhile, it has been demonstrated that commensal microorganisms are able to modulate the expression of IL‐17 and IL‐22 (Figure 2,(7)), which in turn stimulates innate mechanisms that maintain mucosal integrity, such as the production of AMPs and mucins and tissue repair, and then take part in the regulation of the gut microbiota [48]. Moreover, TGF‐β signaling was found to contribute to controlling the gut microbiota composition and maintaining gut barrier integrity by enhancing IgA secretion into the lumen (Figure 2,(8)) [47]. To sum up, the above‐mentioned results proved that intestinal cytokines could be involved in microbiota composition regulation in different pathways, while there were more potential differential pathways where cytokines were involved in the gut microbiota regulation and need further investigation.

### CD4^+^ effector T‐cell and innate lymphoid cells

Moreover, the roles of two immune cell types, cluster of differentiation 4^+^ (CD4^+^) effector T‐cell and innate lymphoid cells (ILCs), in regulating the gut microbiota have recently drawn significant attention. A variety of functionally distinct CD4^+^ T cells exists within the best‐studied subsets in mucosal tissues, namely, Forkhead box protein P3 Tregs, T‐helper type 1 (Th1), Th2, Th17, and T‐follicular helper cells. Of these, Th1, Th2, and Th17 are the main subgroups of CD4^+^ effector T cells that are involved in shaping the gut microbiota [49]. After initiating immune system responses to microbial antigens, IFN‐γ and IL‐12 initiate the differentiation of Th1 cells (Figure 2,(9)), which accelerates the removal of pathogens, and IL‐4 initiates the differentiation of Th2 cells (Figure 2,(10)), which are key in helping B cells to produce antibodies and then regulating the gut microbiota [49]. Notably, IFN‐γ and IL‐4 antagonize each other on different levels, and thus Th1 and Th2 development are considered mutually exclusive [53]. Moreover, IFN‐γ could also promote the intestinal epithelium to produce reactive oxygen and nitrogen, which provides niche and nutrients to boost certain gut microbiota (see the section “Electron respiratory acceptors”). The latest subgroup found is Th17 cells, which induce IECs to produce AMPs and tight junction proteins by secreting IL‐17A, IL‐17F, and IL‐22 [54]. Meanwhile, gut Th17 cells are associated with a variety of inflammatory disorders due to the stimulation of gut microbial antigens [55]. Interestingly, the role of gut Th17 cells in the inflammatory reaction depends on the type of microbial antigens. Symbiotic bacteria induce resident steady‐state intestinal Th17 cells to express IL‐17 and IL‐22 cytokines (Figure 2,(7)) but not TNF and IFN‐γ cytokines, which maintain the state of noninflammatory and mucosal immune barrier (by stimulating the secretion of AMPs by IECs). Th17 cells induced by pathogenic bacteria promote the expression of proinflammatory cytokines and induce inflammatory response (Figure 2,(11)) [50]. Further, RAR‐related orphan receptor γ^+^ (RORγ^+^) Tregs constitute the major subset of colonic Tregs, which play an important role in the homeostasis between immune tolerance and inflammatory reaction [56]. Microbial dysbiosis and an increase of inflammatory Th17 cells were observed in the model animal deficient in RORγ^+^ Tregs [57]. Hence, the induction of intestinal Tregs seems to be a hallmark of host‐microbial immune adaptation.

ILCs are more recently discovered innate cell types that develop from an inhibitor of DNA binding‐2‐dependent common lymphoid progenitor and share functional characteristics with differentiated T cells [58]. ILCs can regulate the gut microbiota by producing cytokines. Overall, how the intestinal CD4^+^ T‐cell compartment reacts to changes in the microbiota composition and whether CD4^+^ T‐cell subset plasticity plays a role in the adaptation to changes in the microbiota composition are currently under investigation. Microbial colonization impacts various immune cells present in the intestine. There is thus a complex interplay within the tissue microenvironment whereby cytokines secreted by one cell type further impact the effector function of other cell types, and in turn, immune mediators can also feed back to and impact the gut microbiota.

## SOURCES RELATED TO GUT‐DERIVED MUCOSAL MOLECULES

The host‐derived nutrients and alternative respiratory electron acceptors from the mucus layer provide an ecological niche on the mucosal layer for microbial growth. Hence, the competition between symbiotic bacteria and pathogens for niches determines the host's resistance to pathogen infection.

### Carbon sources

In monogastric animals, dietary sugar is utilized in the small intestine, which means that microorganisms colonizing the hindgut only use the host glycan and dietary fiber as carbon sources [59]. It is worth noting that ruminants use the forestomach to ferment dietary fiber, which highlights the significance of host glycan for the regulation of the gut microbiota. Here, we mainly discuss the host‐derived carbon sources (host glycan), such as fucose, galactose, and sialic acid, which all originate from the mucus layer of the intestinal epithelium [60]. Therein, fucose is a hexose and lacks a hydroxyl on the sixth carbon atom, which shows stronger hydrophobicity [61]. Sialic acid is a derivative of nine‐carbon sugar and is generally located at the end of glycoconjugates, such as glycoproteins and glycolipids [62]. When referring to detailed mechanical examples, antibiotic treatment can increase host‐derived free sialic acids. The *Bacteroides thetaiotaomicron* (*B. thetaiotaomicron*) has the ability to decompose, but not utilize, sialic acids into a lumen. Free sialic acid provides carbon sources for pathogen growth, which leads to increased host susceptibility to infection [63] (Figure 3,(1)). The host‐derived carbon sources are closely associated with the host genotype. The 2.3‐kb deletion in the *N*‐acetyl‐galactosaminyl‐transferase gene underpinning the ABO blood group decreases the concentrations of *N*‐acetyl‐galactosamine (GalNAc), thereby reducing the abundance of Erysipelotrichaceae that utilize GalNAc as carbon sources in the gut [64] (Figure 3,(2)). On the other hand, the host also has strategies to avoid the utilization of niches by pathogens. For example, the section on immune regulation discussed the capacity of sIgA to increase the polysaccharide utilization activity of symbiotic bacteria, which could increase the mucosal niche competitiveness of beneficial bacteria (see the section “Immune”; Figure 1A,(4)) [27]. Moreover, changing the molecular structure of the host glycan is another strategy. *B. thetaiotaomicron* could also release fucose from mucus to the lumen, which would increase host susceptibility to infection with *S. Typhimurium* [63]. Interestingly, IL‐22, secreted by activated T cells, is one of the inherent defense mechanisms of the host [70] that stimulates the epithelial expression of galactoside 2‐α‐l‐fucosyltransferase2 (FUT2) and the α(1,2)‐fucosylation (glycosylation), which could then not be cut in the mucus layer by *B. thetaiotaomicron* and hence not be utilized as a carbon source by *S. Typhimurium* (Figure 3,(3)) [71].

### Nitrogen sources

The mucin layer of the host, which includes transmembrane and gel‐forming mucins, is also an important nitrogen source for some gut microorganisms (Figure 3,(4)). After an intravenous injection of stable isotope‐labeled threonine (^13^C‐ and ^15^N‐labeled), significant ^13^C and ^15^N labeling was detected in a large subset of the cell population of *Bacteroides acidifaciens* (*B. acidifaciens*) and *Akkermansia muciniphila (A. muciniphila)*, which were proved to be the main foragers using the host‐derived mucin protein [65]. *A. muciniphila* is a mucin‐degrading bacterium of the phylum Verrucomicrobia and its abundance has been shown to be inversely correlated to several disease states, including inflammatory bowel disease (IBD), acute appendicitis, and obesity. *A. muciniphila* has a unique niche advantage in the intestinal mucosal layer, which not only enhances the innate immune response and the gut barrier function but also inhibits the colonization of pathogens in the mucosal layer [72].

### Oxygen sources

A concept of oxygen metabolism and oxygen barrier shaping gut microbiota composition has been recently proposed [66]. In IBD, an increase in the luminal oxygen level could result from the leakage of the epithelium, triggering the release of hemoglobin‐carrying oxygen in the mucus layer where the gut bacteria reside. The increased oxygen level disrupts epithelial anaerobiosis. This could further provide an ecological selective advantage to facultative anaerobes or potential aerobes, which allows them to be more competitive in expanding (Figure 3,(5)). For instance, the aerobic expansion of pathogenic bacteria such as *Salmonella* was found under the disruption of anaerobiosis [73]. Importantly, it was observed that the increase in the luminal oxygen level not only resulted from the leakage of the physical barrier that controls the paracellular pathway but also from the increased anaerobic glycolysis that reduces the oxygen consumption in the transcellular pathway, especially in the colonic epithelia. Unlike the small intestinal epithelia that prefer the usage of glucose and glutamine, the matured colonic epithelia mainly generate energy by oxidizing short‐chain fatty acids such as butyrate, which could render the mucosal surface hypoxic [73]. However, if colonic epithelial cells switch to a preferred use of glucose, the remaining oxygen could diffuse into the intestinal lumen and eventually cause the expansion of facultative anaerobes such as *Enterobacteriaceae*. Indeed, newborn infants have an aerobic intestine at birth [74]. The relatively higher level of oxygen in newborn infants' intestinal tract favors the presence of facultative anaerobes such as *Enterobacteriaceae*, *Enterococcus*, and *Streptococcus*. These early colonizers consume the available oxygen and thereby create an anaerobic microenvironment in the gut and facilitate the formation of obligate anaerobes such as *Bifidobacterium*, *Clostridium*, *Bacteroides*, *Veillonella*, *Eubacterium*, and *Ruminococcus* species. All these evidence support that the oxygen level can shape the regulation of the gut microbiota in the host.

### Electron respiratory acceptors

Another gut‐derived metabolite is the electron respiratory acceptor, which provides conditions for both the aerobic and anaerobic respiration of facultative anaerobic bacteria. Increased oxygen levels in the intestine are associated with inflammatory response and antibiotic treatment, which is unfavorable for the anaerobic microenvironment. However, anaerobic nitrate respiration and sulfate respiration are common strategies for pathogens to colonize the intestine. The proinflammatory cytokine, IFN‐γ, could activate the *Nos2* gene of the intestinal epithelium to produce reactive oxygen and nitrogen (e.g., nitric oxide), which have antibacterial effects but would form nitrate in the lumen [75]. Most members of *Enterobacteriaceae* express nitrate reductase genes, which couple the reduction of nitrate to energy‐conserving electron transport systems for anaerobic respiration, a process referred to as nitrate respiration (Figure 3,(6)) [67]. Interestingly, pathogenic *Salmonella* could not utilize the epithelial‐derived nitrate in the niche, whereas could utilize the nitrate derived from phagocytic infiltrates [76]. It is worth noting that obligate anaerobic symbiotic microorganisms in the intestine cannot express reductase genes [76], which provides niche advantages for facultative anaerobic pathogens. Moreover, the bacteria could adhere itself to enterocytes and hijack the function of these host cells with the help of a syringe‐like apparatus known as a Type III secretion system (T3SS). Depending on the T3SS, the bacteria could utilize the gut‐derived mucosal nutrient (e.g., ethanolamine and fucose) and prevent the unnecessary expenditure of energy [77]. However, *S. Typhimurium* can not utilize ethanolamine under homeostasis or in vitro conditions. The antibiotic streptomycin can activate the oxidation of endogenous sulfur compounds (thiosulfate) to form tetrathionate, the latter acting as an electron respiratory acceptor to enhance the ability of *S. Typhimurium* to utilize ethanolamine (Figure 3,(7) and (8)) [68]. Although the ability to use alternative electron respiratory acceptors can provide conditions for the colonization of facultative anaerobic bacteria, the host could also use this ability to limit the contact of anaerobic symbionts with the intestinal epithelium during homeostasis. *Citrobacter* species (pathogens) could grow in the noninflamed gut through anaerobic hydrogen peroxide (H_2_O_2_) respiration. While H_2_O_2_, produced by NADPH oxidase 1 (*Nox1*) of IECs, could contribute to the inner mucus layer largely devoid of bacteria, which could avoid mucus layer disruption and anaerobic bacterial penetration into crypts during homeostasis (Figure 3,(9)) [69]. It was observed that the regulatory effect of the electron respiratory receptors on the gut microbiota depended on the physiological state of the gut (pathogen infection or homeostasis).

To sum up, host‐derived mucosal metabolism‐related sources could impact the bacteria in terms of their composition in the gut. When discussing host epithelial factors involved in gut microbiota regulation, the epithelial physical barrier with tight junctions and microvilli were often discussed, but their roles in regulating the microbiota is mainly attributed to the physical barrier function. Notably, the destruction of host tight junction and microvilli results in exposure to the environment within the host intestinal epithelium and thereby affects the gut microbiota through gut‐derived mucosal metabolism‐related sources, including the intestinal carbon, nitrogen, and oxygen sources [78].

## GUT‐DERIVED EXOSOMAL NCRNA REGULATION

Exosomes, actively secreted by almost all cell types through an exocytosis pathway, establish the crosstalk between the host and the gut microbiota. The components of exosomes mainly include proteins, lipids, long ncRNA (lncRNA), microRNAs (miRNAs), and circular RNAs (circRNAs) [79]. Of these, exosome proteins and lipids may, respectively, affect the gut microbiota by affecting the carbon and nitrogen sources in the gut. Hence, we propose that more studies focused on the roles of host‐derived exosome proteins and lipids in regulating the gut microbiota are needed. In comparison, more studies about the effects of the components of host‐derived exosomal ncRNAs on the gut microbiota were reported, and we focus on the effects of components of host‐derived exosomal ncRNAs in this part.

### MiRNAs

miRNAs that affect the growth of the microbiota of the lumen mainly originate from IECs and homeodomain‐only protein homeobox (HOPX)‐positive cells. Host‐derived exosomes can carry miRNAs, which could be used as a mediator to realize cross‐border communication between prokaryotic and eukaryotic cells. miRNAs could regulate bacteria gene expression through a combination of messenger RNA (mRNA) destabilization and the inhibition of posttranscriptional regulatory proteins [80]. After miRNAs enter into pathogens (e.g., *Fusobacterium nucleatum* and *E. coli*) by membrane fusion between bacteria and exosomes, they specifically regulate bacterial gene transcripts (Figure 4,(1) and 4,(2)) [81]. Mice with miRNA deficiency exhibited uncontrolled gut microbiota and exacerbated colitis, which could be alleviated by fecal miRNA transplantation [81]. Notably, high‐temperature‐treated fecal transplantation could also have a therapeutic effect because miRNA is heat‐resistant. Besides regulating pathogens, the host‐derived miRNA could regulate symbiotic bacteria. In colitis‐tolerant mice, stimulation with dextran sulfate (DSS) induced IECs to secrete miR142a‐3p, which specifically promoted the growth of *Lactobacillus reuteri* (*L. reuteri*) by combining with specific targets of (locus tag LREU_RS06530 [polA] and locus tag LREU_RS03575) of *L. reuteri* (Figure 4,(3)) [82].

### CircRNA

Both miRNAs and circRNAs can be secreted via exosomes, which have a potential impact on the gut microbiome and health [83]. Zhu et al. [84] reported that microbial colonization in germ‐free mice (specific pathogen‐free microbiota or *Bifidobacterium*) affected the expression of circRNAs in blood and the lung tumor microenvironment. Specifically, the expression of circ0000730 in blood was downregulated when germ‐free mice either received SPF microbiota via fecal transplantation or were inoculated with *Bifidobacterium* [84]. Target prediction analysis revealed that circ0000730 can sponge miR‐466i‐3p and miR‐466f‐3p, which modulate the functions of the SOX9 gene (a developmental regulator and an oncogene) [84]. Therefore, the authors proposed that the circRNA–miRNA–mRNA axis (circ0000730–miR466i‐3p–SOX9 axis) suppresses lung tumors in response to the gut microbiota [84]. This concept adds another layer to the complexity of the host–microbe crosstalk by revealing the interactions among the regulatory circRNAs. Further, Chen et al. [85] used the overexpressed circNF1‐419 adeno‐associated virus animal system to show that the overexpression of circNF1‐419 in the brain not only influenced the brain's cholinergic system but also changed the gut microbiota composition, intestinal homeostasis, and physiology, and the gut microbiota trajectory in newborn mice. Their findings demonstrate a link between circRNAs and the gut microbiome, enlarge the “microbiome–transcriptome” linkage library, and provide more information on the gut–brain axis [86]. To date, research on circRNAs and gut microbiota has been very limited. As negative regulators of miRNAs, circRNAs need to be thoroughly investigated to understand circRNA–miRNA–microbiome interactions (rather than focusing only on the circRNA–microbiome axis). Furthermore, it is equally important to understand the impact of circRNAs on the expression of their target miRNAs in varying tissues and overall host health in response to the gut microbiota and to understand whether circRNAs maintain bidirectional interactions with the host‐associated microbiome, similar to miRNAs.

### LncRNA

In addition to miRNAs and circRNAs, lncRNAs are a group of ncRNA molecules with various lengths and structures. lncRNAs are highly diverse and can connect between gut microbiota and organs other than the gut, for example, gut–placenta axis [87], gut–lung axis [8], and gut–brain axis [88]. Meanwhile, mice that were recolonized with different *E. coli* strains or fecal‐derived microbiota could be discriminated by lncRNA expression profiles of IEC [89]. Hence, lncRNA showed their possibility to interact with the gut microbiota. However, further investigation to decipher the role of lncRNA in microbial regulation is of great importance.

## MOLECULES DERIVED FROM ORGANS OTHER THAN THE GUT

### Hormones from sexual gonads

Gonads regulate the physiological activities of the host mainly by secreting steroid hormones (sex steroids), chiefly including estrogen and androgen, which are closely associated with the dimorphism of diseases.

#### Estrogen

In mammals, estrogens are secreted mainly by the ovaries and placenta, including estradiol (17β‐estradiol), estrone, estriol (16‐hydroxyestradiol), and estetrol (15α‐hydroxyestriol). The gut microorganisms, known as estrobolome, can produce β‐glucuronidase, which decouples conjugated estrogen into an active form [90]. The review pointed out that estrobolome was associated with a variety of diseases (e.g., Alzheimer's disease, osteoporosis, endometriosis, polycystic ovary syndrome, and cancer) by regulating estrogen levels. In particular, women are more susceptible to these diseases after menopause [91], which highlights that the regulatory effect of estrogen on these diseases is inseparable from the role of gut microbiota. However, it may be one‐sided from the perspective of the gut microbiota influencing estrogen levels. Some recent studies have found that estrogen could also affect women's susceptibility to disease by regulating microbial composition.

Estrogen regulating the gut microbiota needs to bind to estrogen receptors (ERs) in intestinal tissue, in which the most classic receptors are receptors α (ERα) and β (ERβ). Estrogen has different regulatory effects on the gut microbiota through ERα and ERβ. The mice with ERα knocked out have a greater tolerance for DSS‐induced colitis, which suggests that ERα could activate a proinflammatory cascade in the intestine (Figure 5A,(1)) [92]. It is worth noting that an inflammatory response could increase the colonization possibility of pathogens and the host susceptibility to diseases (see the section “Sources related to gut‐derived molecules”). On the contrary, the ERβ could regulate differentiation, tight‐junction formation, and permeability of the intestinal epithelium [93]. *Helicobacter hepaticus* (*H. hepaticus*) is a pathogen of the murine intestine that causes intestinal inflammation. In *H. hepaticus*‐infected mice, signaling through ERβ, but not ERα, significantly reduced cecal inflammation as well as the expression of inflammatory cytokines and chemokines [100]. Hence, host susceptibility to intestinal inflammation may be related to the type of ER in the intestinal epithelium. Indeed, studies have found that estrogen could regulate the gut microbiota by binding to the ERβ. Compared with ERβ^+/+^, the abundance of Proteobacteria significantly increased in ERβ^−/−^ mice under the same diet conditions of complex nutrients [101]. Another study also revealed that compared with ERβ^+/+^ mice, α‐diversity of ERβ^−/−^ mice gut microbiota undergoe a greater decline after DSS induction (Figure 5A,(2)) [102]. The G‐protein‐coupled receptor 30 (GPR30) is also one of the specific receptors of estrogen, which is different from ERα and ERβ and resides in the cell membrane. GPR30 could increase colonic motility and may counteract inflammation (Figure 5A,(3)) [95]. Thus, these data hint that estrogen could maintain the stability of gut microbiota and improve the host's tolerance to pathogenic infection through its receptors.

Estrogen is closely associated with susceptibility to infection and gut microbiota. Moreover, estrogen could regulate the gut microbiota by affecting the metabolism of IECs. A study found that Proteobacteria and LPS in the intestine of ovariectomized mice increased significantly, which may be due to estrogen stimulating the production of alkaline phosphatase in the small intestine [103]. Another study identified differences in the expression of AMP genes by comparing the colonic transcriptome of male and female mice [22]. Interestingly, assessable databases [104] reported that the promoter region of some AMPs contains estrogen‐response elements. The above studies suggest that one of the host‐regulating gut microbiota pathways is estrogen–ER–AMP–gut microbiota (Figure 5A,(4)).

#### Androgen

Compared with females, males have a higher prevalence of contracting metabolic diseases. For example, male mice have lower insulin sensitivity than female mice and are more susceptible to high‐fat‐diet‐induced obesity and metabolic syndrome [105]. Apart from estrogen, the sex dimorphism of the disease may be driven by androgens. Androgens mainly include androstenedione and testosterone, which are principally synthesized in the testes as opposed to the adrenal cortex and ovaries in women. In the male and female gonads, the adrenal cortex, and nonendocrine tissues such as in the intestine, circulating androgens can be converted into estrogens by the enzyme aromatase [106].

Interestingly, unlike estrogen, androgen seems to exhibit mixed results in metabolic diseases caused by gut microbiota. For example, adult female mice have a lower gut microbial diversity with a high Firmicutes to Bacteroidetes ratio after neonatal androgen exposure [107]. A recent study revealed the mechanism by which androgens affect gut microbial metabolism. The gut microbiota of castrated male mice was closer to that of female mice in addition to having increased insulin sensitivity. Further, androgen decreased the circulation of glutamine, which could increase insulin sensitivity in vitro. It is worth noting that the effect of androgens on glutamine partially depended on the gut microbiota (Figure 5B,(1)) [108]. However, the effects of androgens on the gut microbiota are not all negative. CRAMP expression was also lower in nonobese diabetic (NOD) female mice compared with NOD male mice, suggesting a protective role of colonic CRAMP against diabetes since male NOD mice are partially protected against the disease (Figure 5B,(2)) [22]. Thus, the effect of androgen on the gut microbiota may depend on specific conditions (e.g., age and disease type), which deserve the attention of researchers in related fields.

#### Neurohormones from the nervous system

Neurohormones are secreted from neuroendocrine cells in response to a neuronal input. Although they are secreted into the blood for a systemic effect, they can also act as neurotransmitters. Meanwhile, the neurohormones are also important host‐derived molecules for the regulation of the gut microbiota. Their effect on the gut microbiota mainly regulates the growth of bacteria and gene expression. For example, catecholamines can alter growth, motility, biofilm formation, and/or the virulence of bacteria (e.g., *Staphylococcus*) [109]. In response to host adrenaline, *Salmonella* downregulates its resistance to host antimicrobial peptides and induces key metal transport systems, which affect the oxidative stress balance in the cells [110]. It could be seen that compared with other host‐derived molecules, neurohormones mainly act directly on microorganisms rather than through the host, and the effect of neurohormones on the gut microbiota has been systematically reviewed by Neuman et al. [111].

### BA from the liver

Cholic acid and chenodeoxycholic acid are produced by the liver with cholesterol as raw material, and then the liver conjugates BAs with glycine or taurine in a two‐step reaction, which produces the primary BA. After entering the small intestine, the primary BA forms secondary BAs under the deconjugation (removal of the glycine or taurine conjugate) of the gut microbiota, 95% of which are reabsorbed back to the liver from the intestine, a process referred to as the enterohepatic circulation of BA [112]. It could be seen that the gut microbiota plays an important role in the circulating concentration of BA, which is closely associated with lipid metabolism. However, the interaction between the gut microbiota and BA is not unidirectional. On the one hand, the BA could promote the growth of BA utilization bacteria but inhibits BA‐sensitive bacteria by destroying the membrane structure (Figure 5C,(1)) [96]. On the other hand, BA could interact with IECs to shape the gut microbiota. BA could induce the transcription of antimicrobial agents (e.g., RNase and nitric oxide) that affect the gut microbiota via the farnesoid X receptor of the small intestine (Figure 5C,(2)) [97]. In fact, a study found that BAs have a profound effect on the immune function of IECs, which further regulates the gut microbiota. The derivatives (3‐oxo lithocholic acid (LCA) and isoalloLCA) of lithocholic acid could be regarded as a T‐cell regulator, in which 3‐oxoLCA could inhibit Th17 cell differentiation and isoalloLCA could enhance Treg differentiation [98]. It is worth noting that the differentiation of Th17 cells could derive the gut microbiota changes (see the section “CD4^+^ effector T cell and ILCs”). Further, the intestinal BA pool could modulate colonic Treg homeostasis and ameliorate host susceptibility to inflammatory colitis via BA nuclear receptors (vitamin D receptor) (Figure 5C,(3)) [113]. It could be observed that the intestinal BA pool plays an important role in the Treg cell homeostasis, which affects the host's susceptibility to infection.

### Lactic acid from the muscle

For the host, the vast majority of lactate processing occurs in the liver. A study found that serum lactate from the liver could cross the epithelial barrier into the lumen of the gut by using ^13^C_3_‐labeled lactate in mice [99]. Interestingly, the long‐term high‐lactate environment of elite athletes could create a dominant niche of gut for *Veillonella atypica* (*V. atypica*) growth. *V. atypica* could metabolize lactic acid to propionic acid, which provides more energy for the body during exercise [99] (Figure 5D). Hence, the lactic acid from the muscle movement could be potential molecule for host‐regulating gut microbiota.

## INTERACTION AMONG DIFFERENTIAL HOST‐DERIVED MOLECULES

Host and gut microbiota form complex networks and it is important to consider the relationship between various host‐derived molecules. For example, IL‐33, TGF‐β, IL‐21, and IL‐4 could increase the secretion of IgA by stimulating B cells [46, 47, 48]; IL‐22, IL‐17, IL‐18, and IL‐1β could trigger the secretion of AMPs by stimulating Paneth cells [41, 42]. IL‐22 could stimulate the secretion of FUT2 and promote the glycosylation of fucose to prevent fucose from being used by pathogens [114]. Moreover, estrogen and androgen from reproductive organs could affect the secretion of AMPs from Paneth cells [22]. Meanwhile, the mechanisms are closely associated with the function of IECs. Most metabolic molecules shape gut microbiota by IgA and AMPs secreted by IEC, which served as an important mediator between host and gut microbiota.

### CLINICAL INSIGHTS OF HOST‐DERIVED MOLECULES ON GUT MICROBIOTA

Expanding knowledge, especially on how host factors shape the gut microbiota, could provide great opportunities to manipulate the gut microbes based on the the individual situation, which has tremendous application potential in the clinic and industry.

It is generally believed that gut microbiota dysbiosis (reducing microbial diversity and richness) increases the susceptibility to infections [115]. Moreover, gut microbiota dysbiosis was closely related to the gut microenvironment changes, which are established and influenced by host‐derived metabolic molecules [116]. For example, reduced luminal IgA secretion in patients with cirrhosis could increase susceptibility to bacteremia and spontaneous bacterial peritonitis [117]. Moreover, decreased expression of colonic RegIIIγ was also associated with increased susceptibility to DSS‐induced colitis [118]. For nongut‐derived metabolic molecules, a decrease in estrogen levels in the body led to gut microbiota dysbiosis, which increases the susceptibility to metabolic syndrome [103]. Hence, identifying the host factors that maintain the homeostasis of gut microbiota provides additional solutions to reduce the risk of diseases such as pathogen susceptibility.

Fecal microbiota transplantation (FMT) is a novel approach to transfer the entire gut microbiota to recipients to restore the gut microbiota homeostasis and treat diseases related to dysbiosis [119]. A meta‐analysis study showed that the cure rate of FMT treatment for *C. difficile* infection (CDI) was higher than conventional treatments. However, the failure rate for FMT to treat CDI patients is 5.2% (colonoscopy)–21.9% (nasogastric tube) [120], which casts a shadow on the practice of FMT. In addition to the FMT dose and frequency, administration method, host factors of the donors, and recipients are also crucial. A study showed that the therapeutic effect of FMT was reflected by the host‐derived molecules that were carried (e.g., miRNA) from the donor [82]. Defined ingredients that have therapeutic effects on donors will reduce the adverse result of treatment. Furthermore, host‐derived molecules in the gut ecological niche of recipients could also decide whether fecal microbiota from donors are colonized and function.

### FUTURE WORK

In this review, we summarized in detail the host‐derived metabolic molecules that shape the gut microbiota, which is closely related to the host genetics (genotype). mGWAS could be used to identify a series of genes associated with the colonization of the microbiota [121]. Moreover, bidirectional Mendelian randomization could use the host genetic variation information obtained through mGWAS as a tool to explore the causality between the host genome and gut microbiota [122]. More tools like mGWAS provide supportive information for researchers or clinicians to predict the host‐derived metabolic molecules that could mediate gut microbiota under host genetic control in the future. When the correlation between host genetic and gut microbiota is established and validated, the researchers or clinicians could evaluate the gut microbiota colonized therein by host genotype. Crohn's disease could be classified into different disease subtypes based on the host genotype and gut microbial compositions [123]. In face of different disease subtypes, clinicians should adopt personalized treatment to maximize the success rate of treatments. Notably, a well‐designed clinical cohort study is needed and strongly suggested to be considered, especially with a large number and longitudinal samples, to assess the effectiveness of special microbial therapy for different disease subtypes diagnosis based on host genotype and gut microbial composition.

To investigate the causality between the host‐derived molecules and gut microbiota, proper experimental models are in great need. Recently, the development of organoids‐on‐chips models provides a solution for considering multiple cell types and gut microbiota. For example, cocultures of microbes, probiotics, or invasive bacteria allow for a gnotobiotic environment in which microbiome and immune elements can be included to see the intersection of multicellular interactions [94]. Moreover, the host cells and bacteria can be cultivated under different oxygen and nutrition concentrations in the same system, which fulfilled the different demands of cells and bacteria [124]. However, further investigations on mechanisms and in vitro models will be essential and of great significance for the field.

Gut microbiota resilience is defined as the ability of gut microbiota to recover from external stress factors such as dietary changes, pathogen infections, inflammatory reactions, and antibiotic therapy [125]. Researchers have used the stability landscape framework to simulate the disturbance of antibiotics to gut microbiota and the subsequent recovery of gut microbiota [126]. A study based on 1000 western adults demonstrated that the abundance of robust bacteria was affected by host factors such as aging and overweight, but not short‐term dietary interventions [127]. In future studies, host factors that could influence the temporal dynamics of gut microbiota, especially the ones disturbed by external factors, need to be explored.

## CONCLUSION

In this review, the molecular mechanisms of how host factors regulate the gut microbiota were summarized. Although a great number of studies have already linked host susceptibility to diseases with the gut microbiota and eating behavior, the precise mechanisms through which the host influences the gut microbiota of some diseases have not yet been fully deciphered. Understanding how host factors regulate the gut microbiota and influence disease incidence may be important to develop novel preventive and therapeutic interventions and even aid in the prediction of disease susceptibility in individuals. It should be noted that the mechanisms mentioned in this review do not exist alone; there is an upstream and downstream regulatory relationship between the mechanisms. Hence, in the face of scientific problems about gut microbiota disorder, researchers need to focus on the genetic differences of intestinal tissues, rather than just the gut microbiota. To date, our understanding of host genetics in modulating gut microbiota has been mainly based on in vitro studies and rodent models. Regarding this, it will be essential to conduct well‐designed clinical trials or assemble clinical data to fill the large gaps between clinical and experimental knowledge and translate the proof of concept acquired from animal models to the clinical setting. Consequently, these studies may potentially be applied for probiotics, prebiotics, and application in the effective treatment of gut microbiota disorder‐related diseases in the future.

## AUTHOR CONTRIBUTIONS

**Chenguang Zhang**: Conceptualization; resources; writing – original draft; writing – reviewing and editing. **Huifeng Liu**: Conceptualization; resources; writing – original draft. **Lei Sun**: Writing – reviewing and editing. **Yue Wang**: Writing – reviewing and editing. **Xiaodong Chen**: Writing – reviewing and editing. **Juan Du**: Supervision; writing – reviewing and editing. **Åsa Sjöling**: Supervision. **Junhu Yao**: Funding acquisition; supervision; writing – reviewing and editing. **Shengru Wu**: Conceptualization; resources; supervision; funding acquisition; writing – reviewing and editing.

## CONFLICT OF INTEREST STATEMENT

The authors declare no conflict of interest.

## Data Availability

The present manuscript is a review paper, so no data are available. Supporting Information: Materials (figures, tables, scripts, graphical abstract, slides, videos, Chinese translated version, and updated materials) may be found in the online DOI or iMeta Science: http://www.imeta.science/.
